# A novel IGSF1 mutation in a large Irish kindred highlights the need for familial screening in the IGSF1 deficiency syndrome

**DOI:** 10.1111/cen.13827

**Published:** 2018-10-01

**Authors:** Edna F. Roche, Anne McGowan, Olympia Koulouri, Marc‐Olivier Turgeon, Adeline K. Nicholas, Emmeline Heffernan, Ranna El‐Khairi, Noina Abid, Greta Lyons, David Halsall, Marco Bonomi, Luca Persani, Mehul T. Dattani, Mark Gurnell, Daniel J. Bernard, Nadia Schoenmakers

**Affiliations:** ^1^ Department of Paediatric Growth, Diabetes and Endocrinology National Children's Hospital Tallaght University Hospital Dublin Ireland; ^2^ Trinity College Dublin The University of Dublin Dublin Ireland; ^3^ Wellcome Trust‐Medical Research Council Institute of Metabolic Science Addenbrooke's Hospital and National Institute for Health Research Cambridge Biomedical Research Centre Addenbrooke's Hospital University of Cambridge Metabolic Research Laboratories Cambridge UK; ^4^ Department of Pharmacology and Therapeutics McGill University Montréal Québec Canada; ^5^ Department of Paediatric Endocrinology & Diabetes Royal Belfast Hospital for Sick Children Belfast UK; ^6^ Wellcome Trust‐Medical Research Council Stem Cell Institute Anne McLaren Laboratory, Department of Surgery University of Cambridge Cambridge UK; ^7^ Wellcome Trust Sanger Institute Cambridge UK; ^8^ Department of Clinical Biochemistry Cambridge University Hospitals NHS Foundation Trust Cambridge UK; ^9^ Department of Clinical Sciences and Community Health University of Milan Milan Italy; ^10^ Division of Endocrinology and Metabolism IRCCS Istituto Auxologico Italiano Milan Italy; ^11^ Section of Genetics and Epigenetics in Health and Disease Genetics and Genomic Medicine Programme University College London Great Ormond Street Institute of Child Health London UK

**Keywords:** central hypothyroidism, congenital hypothyroidism, growth, hypopituitarism, IGSF1, pituitary, thyroid

## Abstract

**Objective:**

Loss‐of‐function mutations in *IGSF1* result in X‐linked central congenital hypothyroidism (CeCH), occurring in isolation or associated with additional pituitary hormone deficits. Intrafamilial penetrance is highly variable and a minority of heterozygous females are also affected. We identified and characterized a novel *IGSF1* mutation and investigated its associated phenotypes in a large Irish kindred.

**Design, Patients and Measurements:**

A novel hemizygous *IGSF1* mutation was identified by direct sequencing in two brothers with CeCH, and its functional consequences were characterized in vitro. Genotype‐phenotype correlations were investigated in the wider kindred.

**Results:**

The mutant IGSF1 protein (c.2318T > C, p.L773P) exhibited decreased plasma membrane expression in vitro due to impaired trafficking from the endoplasmic reticulum. Ten hemizygous males and 11 heterozygous females exhibited characteristic endocrine deficits. Ireland operates a TSH‐based CH screening programme, which does not detect CeCH; therefore, genetic ascertainment preceded biochemical diagnosis of moderate CH in five of seven boys as well as their 75‐year‐old grandfather. Clinical features potentially attributable to hypothyroidism were variable; normal free T3 (FT3) and low/low normal reverse T3 (rT3) concentrations suggested that preferential deiodination of FT4 to FT3 may help maintain tissue euthyroidism in some individuals. However, neonatal jaundice, delayed speech or growth, and obesity were observed in seven subjects in whom diagnosis was delayed.

**Conclusions:**

As observed with other IGSF1 mutations, p.L773P results in variably penetrant IGSF1 deficiency syndrome. Our observations emphasize the need for multi‐generation genetic ascertainment in affected families, especially where TSH‐based CH screening programmes may fail to detect CeCH at birth.

## 1 INTRODUCTION

Central congenital hypothyroidism (CeCH) is a rare entity affecting up to one in 16 000 individuals,^1^ and occurs when hypothalamic and/or pituitary pathology results in inadequate thyrotropin (TSH)‐mediated stimulation of the thyroid gland.^2^ Subnormal circulating free thyroxine (FT4) concentrations in CeCH are associated with a failure of compensatory TSH elevation, therefore CeCH evades detection by the TSH‐based UK and Irish neonatal congenital hypothyroidism (CH) screening programmes, and delayed diagnosis may result in adverse auxological or neurodevelopmental sequelae.^3^ Underlying genetic aetiologies for CeCH include mutations in pituitary transcription factors, which usually manifest as multiple pituitary hormone deficits. Additionally, recessively inherited *TSHB* and *TRHR* mutations, or X‐linked mutations in *TBL1X* or *IGSF1* may present as isolated TSH deficiency.^2^, ^4^, ^5^, ^6^

**Figure 1:**
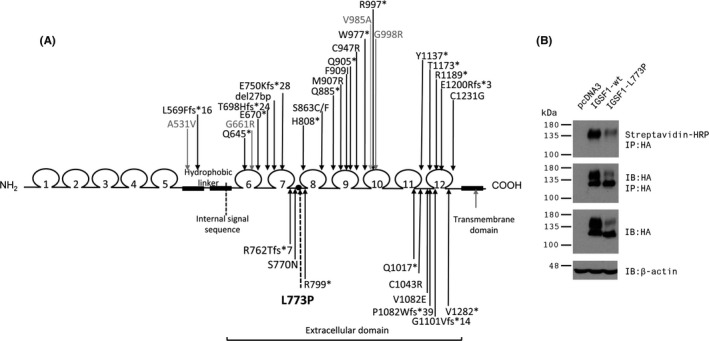
A, Schematic diagram depicting the domain structure of IGSF1 and previously reported mutations. Mutations that either truncate the carboxy‐terminus or have been shown in vitro to exhibit impaired plasma membrane expression are shown in black. Missense mutations that associate with characteristic endocrinopathy, but do not exhibit clear glycosylation or trafficking defects in vitro are shown in grey. The p.L773P mutation is shown in bold with a dashed line. B, In vitro data confirming glycosylation and trafficking defects of the p.L773P mutant protein. HEK293 cells were transfected with empty expression vector (pcDNA3, left lane), or expression vectors for wild‐type (middle lane) or p.L773P (right lane) mutant forms of HA‐tagged IGSF1. Cell surface proteins were biotinylated prior to collection of protein lysates. Proteins were either examined directly by immunoblot (IB) for expression of IGSF1 (HA antibody, third panel from the top) or for β‐actin, used as a loading control (bottom panel), or following immunoprecipitation (IP) with the HA antibody. IP proteins were then examined by IB using HA to confirm precipitation of the IGSF1 protein (second panel from the top) or with streptavidin conjugated to HRP to detect IGSF1 at the plasma membrane (top panel). Molecular weight markers (in kDa) are labelled at the left

Since the initial description of *IGSF1* mutations in eleven European kindreds, larger studies have substantiated the complex nature of the IGSF1 deficiency syndrome as well as confirming the relatively frequent occurrence of *IGSF1* mutations in CeCH cases.^4^, ^7^ *IGSF1* encodes a transmembrane immunoglobulin superfamily glycoprotein that undergoes co‐translational proteolysis such that only its seven carboxy‐terminal immunoglobulin loops are expressed extracellularly at the plasma membrane.^8^ The majority of previously reported *IGSF1* mutations adversely affect trafficking and membrane localization of this carboxy‐terminal domain^9^ (Figure 1A). IGSF1 is abundantly detected at mRNA level in Rathke's pouch and adult pituitary gland^4^; however, a paucity of reliable antibodies has hampered expression studies in humans. In rodents, differential antibody usage has yielded divergent results; IGSF1 protein has been detected in all cells of the Pou1f1 (Pit1) lineage in murine and rat pituitaries using one custom IGSF1‐CTD antibody^4^, ^10^; however, a different, commercially available anti‐IGSF1 antibody (Genetex) localized IGSF1 to thyrotropes and gonadotropes in rats, but not somatotropes or lactotropes.^11^ Despite clinical and murine data supporting a role for IGSF1 in regulation of TRH action in the pituitary, its molecular function remains undefined.^4^, ^12^, ^13^

Hormone deficiencies associated with *IGSF1* mutations may involve all cells of the POU1F1 lineage. Hemizygous males almost universally exhibit central hypothyroidism and 60% have basal hypoprolactinaemia; a minority (~15%) exhibit transient childhood growth hormone (GH) deficiency.^9^ Endocrine evaluation of heterozygous females usually reveals a milder phenotype with FT4 concentrations in the lower tertile of the normal range, although 18% do exhibit overt central hypothyroidism and 22% have subnormal basal prolactin.^7^, ^9^ Affected boys exhibit delayed pubertal testosterone rise and growth spurt, associated with preserved testicular growth and development of macroorchidism from late adolescence onwards.^4^, ^9^ Additional features include raised BMI, and mildly elevated or high‐normal adult IGF‐1 concentrations.^7^, ^9^

Biochemical and physiological severity of CeCH are variable in IGSF1 deficiency (including in congenic mouse strains), and some individuals tolerate lifelong thyroid hormone deficiency without apparent adverse consequences, whereas others present symptomatically early on.^4^, ^9^, ^11^ Here, we describe a large Irish kindred in which two male siblings with CeCH were found to harbour a novel missense *IGSF1* mutation. In vitro evaluation of the mutant IGSF1 protein demonstrated reduced plasma membrane expression. Family screening identified eight additional hemizygous males and 11 heterozygous females who subsequently underwent endocrine evaluation. Tissue manifestations of hypothyroidism reflect the pleiotropic effects of thyroid hormone and in childhood may include growth retardation, delayed bone age or neurodevelopmental milestones, and prolonged neonatal jaundice. Affected children and adults may also exhibit bradycardia, hypothermia, overweight and dyslipidaemia and may describe constipation and fatigue.^14^ Biochemical severity of hypothyroidism was usually moderate in our kindred and some cases appeared to tolerate hypothyroidism remarkably well, whereas seven individuals exhibited adverse sequelae potentially attributable to thyroid hormone deficiency. Our observations support the notion that although some individuals are apparently asymptomatic despite significant central hypothyroidism, family screening in this context remains crucial in enabling prompt diagnosis in cases where growth and development may otherwise be impaired.

## 2 MATERIALS AND METHODS

The study was approved by Cambridge South REC (MREC 98/5/24) and includes additional measurements undertaken as part of routine clinical follow‐up with consent from patients and/or next of kin.

### 2.1 Sanger sequencing of *IGSF1*

Genomic DNA was extracted from peripheral blood leukocytes using standard techniques. In the probands, all 20 *IGSF1* exons and exon/intron boundaries were amplified by PCR using specific primers (available on request). Family members were genotyped for the identified missense mutation (see Results) by amplifying and sequencing exon 13 alone. PCR products were sequenced using the BigDye Terminator v3.1 Cycle Sequencing Kit (Applied Biosystems, Foster City, CA) and 3730 DNA Analyzer (Applied Biosystems). The *IGSF1* variant listed in this study is described using the systematic nomenclature approved by the Human Genome Variation Society (HGVS; http://www.hgvs.org/mutnomen). Nucleotide numbering starts from the A (+1) of the translation initiation codon (ATG) of the NCBI reference sequence NM_001170961.1. Amino acid residues are numbered according to the NCBI reference sequence NP_001164432.

### 2.2 Clinical measurements

All biochemical measurements were performed in CPA (Clinical Pathology Accreditation) and INAB (Irish National Accreditation Board) accredited laboratories, using local automated assays, and results were compared to local reference ranges (age and gender‐specific where appropriate). Auxological measurements were performed by trained auxologists during clinical visits, and SDS scores were computed according to published reference data^15^, ^16^, ^17^ using GrowthXP Endo, a children growth monitoring commercial programme.

Relevant medical history was acquired by review of patients’ notes or direct questioning. Unless otherwise stated, reference ranges refer to 95% confidence intervals, and variance is reported as standard deviation score. Sleeping heart rate was compared to a locally‐generated reference range for healthy males and depicts minimum and maximum. Basal metabolic rate was measured using ventilated hood indirect calorimetry as described in.^18^

### 2.3 TRH tests

TSH, FT4, free triiodothyronine (FT3) and prolactin were measured in serum samples taken at baseline and at 20, 60, 90, 120, 150 and 180 minutes following administration of 200 micrograms of Protirelin (Thyrotrophin‐releasing hormone, TRH, Alliance Pharmaceuticals Ltd, Chippenham, UK). Hormone responses were compared with control subjects aged 18‐67 years (mean 33.8 ± 13.7) from Milan, Italy, including 12 healthy individuals and 9 cases with nonfunctioning microlesions of the pituitary (<5 mm in diameter) without pituitary hormone deficits.

### 2.4 Testicular volume assessment

Calculation of ultrasonographic testicular volume was computed as previously described and compared with published reference data.^19^ Testicular volumes in children were assessed clinically by a trained paediatric endocrinologist using the Prader Orchidometer.

### 2.5 In vitro analysis of the mutant IGSF1 protein

The L773P mutation was introduced into the human myc‐IGSF1‐HA expression vector described in^8^ using the QuikChange mutagenesis protocol and the following primer set: (Forward: GCCCAGTGAGCCGCCGGAGCTTGTCATAA, Reverse: TTATGACAAGCTCCGGCGGCTCACTGGGC). Mutant and wild‐type IGSF1 were expressed in human embryonic kidney (HEK) 293 cells. Transfections, cell surface biotinylation, immunoprecipitations, SDS‐PAGE and immunoblotting were performed as described in.^4^

## 3 RESULTS

### 3.1 Patient histories

**Figure 2:**
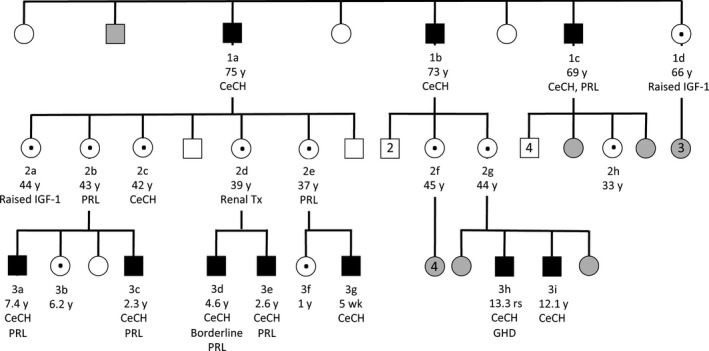
Pedigree of the kindred harbouring the p.L773P mutation. Hemizygous males are shown with black boxes; heterozygous females are shown with a central black dot. Confirmed or obligate wild‐type cases are shown in white; cases who have not been tested, and whose genotype is unknown are in grey. Cases are annotated with ID, age, and hormone deficiencies, including central hypothyroidism (CeCH), hypoprolactinaemia (PRL), GH deficiency (GHD) and increased IGF‐1 concentrations

**Figure 3:**
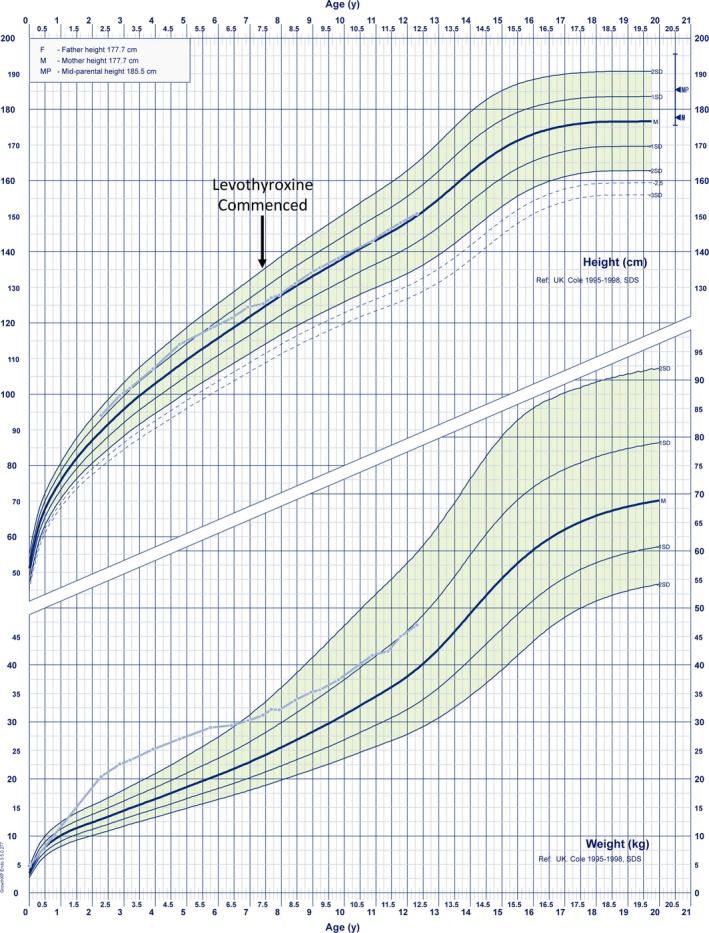
Growth chart demonstrating sequential height and weight SDS in case 3a (pale blue). The arrow denotes commencement of levothyroxine treatment [Colour figure can be viewed at http://www.wileyonlinelibrary.com]

**Figure 4:**
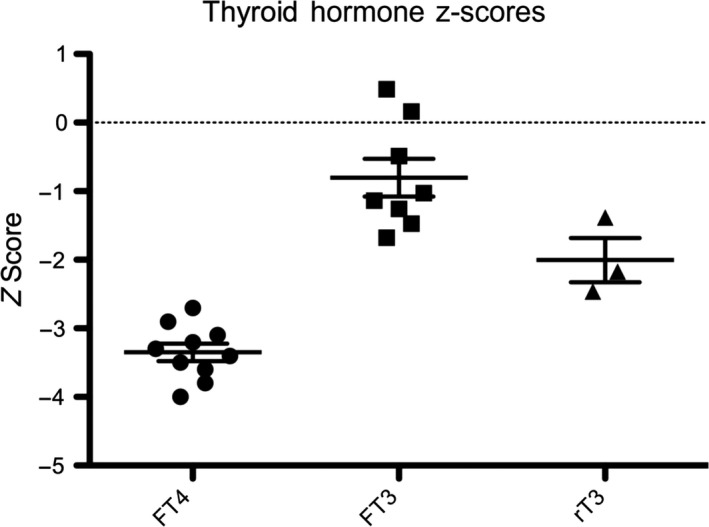
Distribution of FT4, FT3 and rT3 Z‐scores in hemizygous p.L773P cases. Cases exhibit subnormal FT4, normal FT3 and low/low‐normal rT3 concentrations. Black horizontal lines represent the mean value; bars denote standard error of the mean (SEM). The population mean is denoted by the dashed line at 0

**Figure 5:**
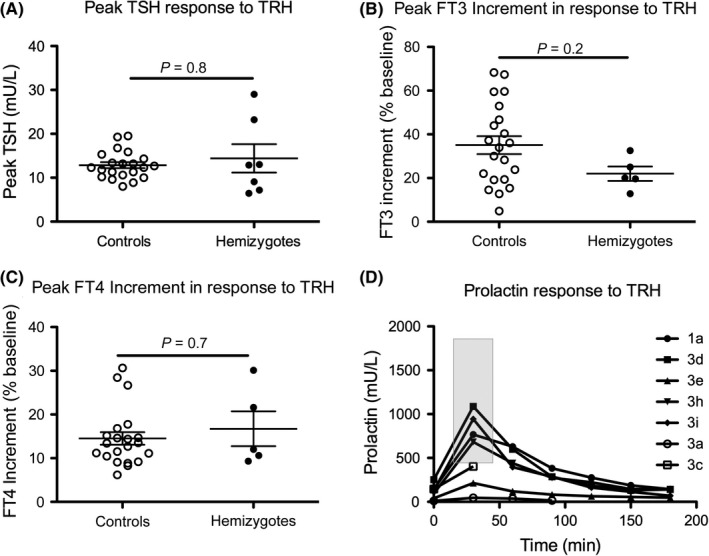
Peak A, TSH, B, FT3, C, FT4 and D, sequential prolactin measurements during a TRH test in 7 (TSH, Prolactin) or 5 (FT3, FT4) hemizygous male cases aged 2.2‐75 years. Peak TSH and prolactin increment occurred at 20 mins in all cases and maximal FT3 increment, which was assessed using measurements up to 180 min after administration of TRH, usually occurred at 150 min (range 120‐180 min). Black horizontal lines represent the mean value; bars denote standard error of the mean (SEM), and *P*‐values were calculated using a Mann‐Whitney U test. The Grey box defines the reference range for prolactin peak in accordance with published literature; RR 421‐1829 mU/L, minimum to maximum 32

The male Proband, 3a (Figure 2), was born at term to nonconsanguineous Irish parents (birth weight 4.7 kg, SDS +2.49) (Figures 2, 3, 4, 5; Tables 1 and 2; Tables S1‐S3). He exhibited mild transient neonatal hypoglycaemia and jaundice, requiring 24‐hour phototherapy. TSH screening results were normal; however, at age 22 months, he was noted to be obese (weight 18.9 kg, SDS +4.09, BMI 23.9 kg/m^2^, SDS +4.0) with an intermittently subnormal FT4 [9.1‐10.5 pmol/L (RR 10‐25)], normal TSH [2.06‐3.5 mU/L (RR 0.4‐4.0)] and undetectable prolactin [<10 mU/L (RR 90‐320 U/L)]. A synacthen test demonstrated a normal cortisol response (basal cortisol 204 mmol/L, 30 minutes 773 mmol/L, 60 minutes 802 mmol/L. As FT4 remained low normal, he was not commenced on levothyroxine. His BMI decreased steadily with dietary intervention and development was normal. However, between age 6.9 and 7.4 years, the previously normal annualized height velocity declined to 1.91 cm/y (height velocity SDS decrease from +0.1 to −4.56) and growth declined from height SDS +0.59 to +0.21 prompting re‐evaluation, which confirmed central hypothyroidism with a subnormal FT4 [10.5 pmol/L (RR 12‐22)], FT3 at the lower limit of normal [3.4 pmol/L (RR 3.1‐6.8)] and inappropriately normal TSH [2.68 mU/L (RR 0.3‐4.2)]. IGF‐1 [13.3 mmol/L (RR 7‐40)] and IGFBP‐3 [3.93 mg/L (RR 1.1‐4.3)] were normal, and pituitary MR imaging was unremarkable. Height SDS remained stable and height velocity improved steadily with levothyroxine replacement (SDS −4.56 aged 7.4 years to +1.12 aged 8.5 years, see growth chart, Figure 3).

**Table 1:** Baseline endocrinology and auxology (hemizygotes)

Case ID	Age years	BW (kg) (SDS)	Height, cm (SDS)	BMI (kg/m^2^) (SDS)	TSH mU/L (0.3‐5.5)	fT4 (pmol/L)	fT3 (pmol/L)	PRL (Z score)	IGF1 (Z score)	Testo (8‐29 nmol/L)	FSH:LH (Adults) (0.4‐3.4)	Hypothyroid Signs
1a	75.5	Estimated BW 4.5 kg	181 (0.66)	**32.1**	1.22	**6.9** (12‐22)	3.9 (3.5‐6.5)	−1.4	1.1	12.7	2.9	Obesity
1b	56.3	NA	175 (−0.2)	**35.9** a	0.97	**8.9** (12‐22)	NA	0.7	−0.5	10.7	2.6	Fatigue, obesity, dyslipidaemia
1c	69.1	NA	172 (−0.63)	**31.8**	1.59	**8.1** (12‐22)	NA	−**2.2**	−0.5	**6.7**	3.2	Fatigue, obesity
3a	7.4	**4.7 kg (+2.49)**	125.4 (+0.21)	**19.8 (+2.09)**	0.89	**10.2** (12‐22)	3.9 (3.1‐6.8)	−**3.4**	−1.2	‐	‐	Transient neonatal jaundice, obesity, (improved with diet) decline in growth velocity aged 7
	2.2		0.9 (+0.36)	**23.95** **(+4.2)**								
3c	2.3	**4.48 kg** **(+ 2.06)**	90.8 (+0.21)	**20.6** **(+2.92)**	2.8	**8.3** (12‐22)	5.1 (3.1‐6.8)	−**2.2**	−1.0	‐	‐	Persistent neonatal jaundice (6 mo), speech delay, obesity
3d	4.6	1.81kg^b^ (−4.32)	109 (+0.71)	16.1 (+0.28)	2.88	**9.3** (12‐22)	4.0 (3.1‐6.8)	−**2.0**	−1.4	‐	‐	Nil
3e	2.6	2.77 kg^b^ (−1.48)	93.7 (+0.71)	18.5 (+1.9)	2.07	**9.7** (12‐22)	4.5 (3.1‐6.8)	−**3.0**	−1.4	‐	‐	Nil
3g	5 wk	**4.5 kg** **(+2.1)**	**50.7** (−2.22)	NA	3.31	**7.6** (12‐22)	5.4 (3.1‐6.8)	0.8	−0.9	‐	‐	Nil
3h^c^	14.2	3.73 kg (+0.54)	**142** (−3.1)	22 (+0.65)	1.38	**9.9** (12.6‐21)	4.2 (3.9‐7.7)	−1.2	−**2.2**	0.3	‐	Growth retardation
3i	12.1	3.58 kg (+0.22)	**135.2** (−1.86)	22.1 (+1.67)	1.69	**9.7** (12.6‐21)	4.6 (3.9‐7.7)	−1.3	−**2.0**	<0.2	‐	Growth retardation, neonatal hypothermia

Normal Ranges are defined in brackets and denote minimum‐maximum for FSH:LH ratio, which is shown for adults only. BW, Birth weight; PRL, prolactin; Testo, Testosterone.

Abnormal values are highlighted in bold. Where multiple sets of thyroid hormone measurements were available, tabulated values refer to a representative set of measurements obtained prior to commencing levothyroxine treatment.

BMI aged 74 y.

Maternal renal transplant prior to pregnancy.

Also had growth hormone deficiency, diagnosed aged 14.5 y, which may have influenced growth.

**Table 2:** Baseline endocrinology (Heterozygotes)

Case ID	Age (y)	TSH (0.3‐5.5 mU/L)	fT4 (12‐22 pmol/L)	fT3 (3.5‐6.5 pmol/L)	PRL (Z score)	IGF‐1 (Z score)
1d	66	2.17	13.2^a^	4.5	−0.9	**2.2**
2a	44.8	1.81	13.4	NA	−1.7	**2.9**
2b	43.8	2.03	12.4	NA	−2.7	NA
2c	42.6	1.08	**11.7**	4.4	−1.3	0.4
2d	39.9	2.54	14.1	NA	0.8	−1.5
2e	37.3	1.9	12.9	NA	−2.3	1.1
2f	45.7	2.87	13.9^a^	4.1	−1.4	−0.4
2g	44.6	1.75	12.8^a^	4.6	−0.7	−0.1
2h	33.9	1.47	16.6	4.5^a^	0.7	1.0
3b	6.2	3.44	15.4	6.3^a^	−2.8	NA
3f	1.0	3.28	14.3	5.1^a^	−1.0	−1.2

Normal Ranges are defined in brackets.

Reference range 10‐19.8 pmol/L.

a Reference range 3.1‐6.8 Abnormal values are highlighted in bold.

His brother, 3c, was born at 42 weeks’ gestation with a birth weight of 4.48 kg (SDS +2.06). Neonatal jaundice was treated with phototherapy at postnatal days 1 and 14 but persisted for 6 months with elevated transaminases. He had mild pulmonary branch stenosis, speech delay and glue ear. At age 2.3 years, he was noted to have a subnormal FT4 [9.1 pmol/L (RR 12‐22)] with a normal FT3 [5.0 pmol/L (RR 3.1‐6.8)] and inappropriately normal TSH [2.73 mU/L (RR 0.3‐4.2)]. Prolactin was low at 79 mU/L (RR 90‐320). He also exhibited increased weight (17 kg, SDS +2.42, BMI 20.6 kg/m^2^, SDS +2.92), but grew along the 75th percentile with a normal height velocity. Levothyroxine therapy was commenced, and he has progressed well at school; his speech is now also normal. The occurrence of familial CeCH with hypoprolactinaemia in one sibling prompted screening of *IGSF1*, which identified a shared, novel missense mutation, c.2318T > C, p.L773P (Figures 1A,B and 2).

### 3.2 Pathogenicity of mutation

The missense variant identified was absent from published databases [dbSNP, Exome Aggregation Consortium (ExAC), Cambridge, MA (URL: http://exac.broadinstitute.org), May 2018] and altered a highly conserved amino acid. To determine the potential functional impact of the p.L773P variant, we expressed the wild‐type and mutant forms of the protein in heterologous HEK293 cells. Wild‐type IGSF1 migrates as a doublet on SDS‐PAGE with the higher molecular weight band representing the mature, plasma membrane glycoform (Figure 1B, centre lane in the middle two panels). The lower molecular weight band corresponds to the immature, endoplasmic reticulum (ER)‐retained glycoform. The p.L773P mutant similarly migrated as a doublet, but with a notable reduction in the abundance of the mature glycoform (Figure 1B, right most lane). Indeed, far less IGSF1‐ L773P was observed at the plasma membrane than wild‐type IGSF1, as assessed by cell surface biotinylation (Figure 1B, top panel). These data indicate that the trafficking of IGSF1‐L773P out of the ER to the plasma membrane is impaired.

### 3.3 Additional genetic ascertainment and endocrine evaluation

Family screening identified an additional eight hemizygous males (five children and three adults) and eleven heterozygous females. Baseline endocrinology and auxology was assessed, and dynamic endocrine testing was performed in a subset of individuals (Figures 2, 3, 4, 5; Tables 1 and 2; Supporting Information Tables S1‐S3).

### 3.4 Thyroid function

All evaluable male cases exhibited central hypothyroidism (FT4 ranging from 6.9 to 10.2 pmol/L, mean Z‐score −3.3 ± 0.4) although biochemical penetrance was variable. In all cases, FT3 concentrations were maintained within the reference range (mean Z‐score −0.8 ± 0.8) despite a subnormal FT4, and in three evaluable cases, this was achieved at the expense of low/low normal reverse T3 concentrations (Figures 2 and 4). Assessment of physiological thyroid status is challenging due to a paucity of sensitive and specific biomarkers; however, we screened our cases for features potentially attributable to tissue hypothyroidism including high birth weight, neonatal complications of hypothyroxinaemia, obesity, developmental delay, growth impairment and symptoms such as constipation and fatigue (Table 1). Three of seven cases for whom data was available (3a, 3c, 3g) exhibited birth weight SDS scores of greater than +2.0 and case 1a was reportedly a “large baby” with an estimated birth weight of around 4.5 kg; cases 3d and 3e exhibited low birth weight SDS which may have been attributable to maternal renal disease. One child and two adolescents (cases 3a, 3h, and 3i) with mild‐moderate central hypothyroidism, exhibited growth retardation or a decline in growth velocity; as previously detailed, 3a exhibited a growth response to levothyroxine (Figure 3) and 3i also exhibited improved growth after twelve months of levothyroxine treatment (height SDS improvement from SDS −1.86 to +1.67); however, his levothyroxine dose continues to be adjusted since his thyroid hormone concentrations remain suboptimal (FT4 12.4 pmol/L, RR 12.6‐21), precluding full assessment of his response. Case 3i had neonatal hypothermia and case 3a had required dietary intervention for obesity (Figures 2 and 4; Table 1). However, three children (cases 3d, 3e, and 3g) were apparently clinically euthyroid; despite moderately subnormal FT4 concentrations. Three adults were genetically ascertained in their 6th‐8th decades. Two cases (1b, 1c) with moderate hypothyroidism had previously been commenced on levothyroxine aged 56 and 59 years after presenting with fatigue and obesity, despite attaining normal height. Case 1a had significant biochemical hypothyroidism at diagnosis aged 75.5 years; [fT4 6.9 (RR 12‐22 pmol/L)] and we selected this patient for more detailed biochemical and physiological assessment. Although obese (BMI 32.1 kg/m^2^), he was otherwise asymptomatic, having attained normal height. Several biochemical markers of thyroid hormone action were normal; cholesterol 3.9 mmol/L, LDL‐cholesterol 2.26 mmol/L, CK 204 (40‐320 mU/L), SHBG 36.3 (10‐57 mmol/L), and his IGF‐1 concentration (a positively‐regulated thyroid hormone target gene) was also inappropriately in the upper part of the normal range given his subnormal fT4 [IGF‐1 25.7 mmol/L, (RR 8.5‐30.7, Z score +1.1)]. Cognitive function was not formally characterized but he had no overt neurodevelopmental abnormalities (Figures 2 and 4; Table 1, Supporting Information Table S1) Basal metabolic rate was normal (7.8 MJ/d), equating to 101.8% value computed using the Schofield predictive equation,^20^ sleeping heart rate was unremarkable (55 beats/min, RR 46‐67, mean 55 beats/min) and clinical examination (warm palms, normal Achilles tendon reflex, lack of dry skin or hypothyroid facies) did not reveal any other features of hypothyroidism. Therefore, although other family members with less marked biochemical hypothyroidism exhibited some features consistent with physiological hypothyroidism, surprisingly, this case did not have readily discernable tissue hypothyroidism despite markedly subnormal FT4 levels.

Only one heterozygous female (case 2c, aged 42.6 years) exhibited central hypothyroidism [TSH 1.08 mU/L (RR 0.3‐4.2); FT4 11.7 pmol/L (RR 12‐22)]. The majority of heterozygotes had FT4 concentrations in the lower third of the reference range (mean FT4 13.7 ± 1.39 pmol/L, mean Z‐score −0.5 ± 0.3) (Table 2).

### 3.5 Prolactin

Prolactin concentrations were usually in the lower half of the reference range (hemizygotes; mean Z‐score −1.5 ± 1.4, heterozygotes; mean Z score −1.2 ± 1.2). One hemizygous adult and three children exhibited hypoprolactinaemia, with prolactin concentrations ranging from undetectable (case 3a) to subnormal (cases 3c, 3e, and 1c) or low‐normal [case 3d, 92 mU/L (RR 90‐320)]. Two heterozygous adults and one child were hypoprolactinaemic; however, both adults experienced no problems with pregnancy although case 2b did experience difficulties with lactation with her two sons (Tables 1 and 2; Supporting Information Tables S1 and S2).

### 3.6 Gonadal axis

The FSH:LH ratio was in the upper part of the reference range in the three male hemizygous adults, mean 2.91 (RR 0.4‐3.4) (Table 1; Table S1). Ultrasonography in case 1a confirmed macro‐orchidism (mean testicular volume 22.3 mL, Z score +2.4) compared with age‐matched reference data.^19^ Two children (3h, 3i) had clinically assessed testicular volumes of 8 mL [(Z score −1.25), 3h, aged 14.2 years] and 15 mL [(Z score +0.25), 3i, aged 13.9 years]. It was not possible to retrieve accurate data regarding pubertal development in cases 1b and 1c. 2a had an ovarian mass thought to be a cyst detected incidentally on ultrasound performed aged 38 (6 years prior to the current study). 2b also had an ovarian cyst reported following abdominal ultrasound for midcycle pain. Histological evaluation of the cysts has not been undertaken and neither patient has undergone surgical intervention.

### 3.7 Growth hormone

Two male cases had either low normal (3i) or mildly subnormal (3h) IGF‐1 concentrations at diagnosis; however, both were evaluated whilst hypothyroid and peripubertal (aged 12 and 13 years); therefore, GH deficiency could not be definitively excluded or confirmed. Case 3i showed improved growth on levothyroxine, but Case 3h was still growing slowly at age 14.5 years (height velocity 4.1 cm/y, height 142 cm, <0.4th centile, testosterone <0.2 nmol/L, pubertal staging G3, P1, A1, testicular volumes 8 mL), despite low normal IGF‐1 (19.7 nmol/L). An insulin tolerance test (ITT) after testosterone priming confirmed growth hormone deficiency (peak GH 1.8 μg/L at 60 minutes). In Case 3a, when thyroxine replete, GH concentrations were insufficient (GH peak of 4.01 μg/L, baseline 3.36 μg/L on ITT post‐testosterone priming at age 11.4 years), at which point testicular volumes were 4 mL bilaterally. However, IGF‐1 (27.3 mmol/L, RR 10.8‐63.7) and IGFBP3 (4.07 mg/L, RR 2.5‐6) were within normal limits, and although the test had been performed due to declining growth velocity, this resolved without intervention. Cortisol response was preserved (peak 529 mmol/L).

Paradoxically, IGF‐1 concentrations were elevated in two of nine adult heterozygous females [Cases 1d, 29.5 nmol/L (RR 11.8‐28.6); and 2a, 32.7 nmol/L (RR 2.7‐28.2)] and were otherwise in the mid‐normal range (mean Z score +0.5 ± 1.5), with the exception of Case 2d who had had renal transplantation following a diagnosis of membranoproliferative glomerulonephritis [IGF‐1, 11.1 nmol/L (RR 8.5‐30.7)]. IGF‐1 concentrations in the hemizygous adult males were in the mid‐normal range (mean Z score 0.04 ± 0.9), but were more variable in the hemizygous male children (mean Z score −1.46 ± 0.46) (Tables 1 and 2; Supporting Information Tables S1 and S2).

### 3.8 TRH testing

A TRH test was performed in seven males [1 adult (Case 1a) and six children (Cases 3a,c,d,e,h,i)]; five cases underwent prolonged testing. The TSH peak occurred at 20 minutes in all cases and was normal or exuberant (mean peak 14.4 ± 8.6 mU/L (RR 12.9 ± 3.1)); however, the maximal increment in FT3 and FT4 (an indirect indicator of TSH bioactivity) was not significantly different to controls, although FT3 increment fell in the lower half of the normal range [22% ± 7.3 (RR 35.1 ± 18.7)]. The two childhood cases exhibiting the more marked TSH responses did not undergo prolonged testing and were therefore not included in this assessment. Peak prolactin was normal in four males with normal basal prolactin (Cases 1a, 3d, h, i) but subnormal in Cases 3a and 3e, who had basal hypoprolactinaemia, although a fivefold increase in Case 3e confirmed some prolactin reserve (Figure 5; Supporting Information Table S3).

## 4 DISCUSSION

We identified a novel IGSF1 missense mutation in a three‐generation Irish kindred. This is the largest reported family to date, with endocrine evaluation of 10 hemizygous males and 11 heterozygous females. Previously described *IGSF1* mutations include four whole‐gene deletions,^4^, ^11^, ^21^ multiple premature truncations and missense mutations, 2 splice‐site mutations and a 27‐bp deletion 4, 7, 9, 22, 23, 24, 25, 26, 27, 28 (Figure 1A). Affected individuals in our kindred exhibit classical endocrine manifestations of IGSF1 deficiency, and characterization of the p.L773P mutation in vitro demonstrates deficits in trafficking of the protein from the endoplasmic reticulum to the cell surface, as has been described for previously‐reported pathogenic *IGSF1* mutations.^4^, ^7^, ^9^

In keeping with previous reports, biochemical penetrance of thyroid dysfunction in the L773P kindred was variable, although all hemizygous males had mild or moderate central hypothyroidism according to ESPE criteria.^29^ Ascertainment following identification of an *IGSF1* mutation may precipitate diagnosis of moderate central hypothyroidism in apparently asymptomatic individuals, whereas other subjects with comparable biochemistry present symptomatically, suggesting variable physiological penetrance.^4^ Consistent with this, Case 1a had the lowest FT4 concentrations (6.9 pmol/L). However, although obese, he had attained normal height, and seemed otherwise physiologically euthyroid when evaluated using detailed biochemical and physiological indicators of tissue thyroid status. In contrast, a subset of individuals (Cases 3h, 3i, 3a, 3c) with biochemically milder disease exhibited features potentially consistent with tissue hypothyroidism. Although associated GH deficiency is a potential confounder in Cases 3a and 3h, it is likely that hypothyroidism was the most significant contributor to growth impairment in 3a, given the improved growth velocity once levothyroxine treatment alone was commenced. Additionally, 3i exhibited a growth response to levothyroxine supporting a hypothyroid aetiology for his short stature Increased BMI is a recognized association of IGSF1 deficiency, and five cases were obese, perhaps also reflecting tissue hypothyroidism^9^ although the specificity of weight gain for thyroid dysfunction is poor. Additionally, birth weight SDS was greater than +2SDS in three of seven evaluable hemizygotes, which is also a recognized feature of congenital hypothyroidism as well as being reported in 25% IGSF1 deficient males.^9^, ^30^

Subnormal FT4 is often associated with normal FT3 concentrations in IGSF1 deficiency, and FT3 was preserved in all p.L773P males at diagnosis. In three evaluable cases, rT3 was also subnormal or low normal, potentially consistent with preferential deiodination of FT4 to FT3. Although normal serum FT3 concentrations did not preclude development of symptomatic hypothyroidism, increased conversion of FT4 to FT3 may modulate tissue hypothyroidism in some subjects.^4^ Alternatively, a history of apparently normal development in hypothyroid adults may reflect evolution of milder childhood hypothyroidism. Indeed, fluctuating thyroid hormone concentrations have previously been reported in IGSF1 deficiency; however, future studies are needed to characterize the natural history of thyroid dysfunction in this syndrome.^25^

IGSF1 is thought to have a role in murine TRH signalling since *Igsf1* null mice exhibit preserved hypothalamic *Trh* expression with decreased pituitary *Trhr1* mRNA levels and subnormal TSH response to exogenous TRH.^4^, ^12^ Human studies generally support a similar function, with subnormal (neonatal) or low‐normal (child‐adulthood) TSH response to TRH in affected males and subnormal FT4 increment following the TSH peak.^4^, ^9^ TRH testing in the hemizygotes reported here elicited normal or exuberant TSH responses, and comparable FT4 and FT3 increments to controls, although FT3 fell in the lower half of the reference range. Impaired post‐translational modification of TSH would be an expected consequence of a TRH signalling defect, and TSH bioactivity was markedly subnormal when assessed directly in one previous case.^11^ However, our findings suggest that TSH bioactivity may only be mildly impaired in some cases of IGSF1 deficiency, although this requires confirmation by direct quantitation in future studies.

Biochemical severity of hypothyroidism did not predict coexistence of other hormone deficiencies; however, three males exhibited subnormal/borderline basal prolactin. In keeping with previous reports, hemizygous cases (n = 4) with normal basal prolactin exhibited a normal prolactin response to TRH, whereas the peak was blunted in the context of basal hypoprolactinaemia (Figure 5, Cases 3a and 3e).^22^, ^23^, ^26^ The role of IGSF1 in prolactin production remains unclear and is likely to involve pathways separate from the TRHR, as TRHR signalling is not required for normal basal prolactin production.^2^, ^31^ Female hypoprolactinaemia had no apparent effect on pregnancy, although one individual experienced difficulties with lactation.

Although IGSF1 expression has been reported in murine and rat somatotrophs, its role in GH dynamics may be complex. GH deficiency occurs rarely in IGSF1 deficiency; however, IGF‐1 is usually in the upper part of normal range in IGSF1 deficient adults and may be associated with acromegaloid features consistent with mild GH excess.^7^, ^9^ In our kindred, one individual had GH deficiency and another had insufficient GH concentrations on stimulation testing with normal IGF‐1 and growth velocity. However, the potential for puberty to be delayed in the context of IGSF1 deficiency may complicate interpretation of these results. Two adult heterozygotes had mildly elevated IGF‐1 concentrations (Cases 1d and 2a) and one hemizygote (Case 1a) exhibited acromegaloid facies with inappropriately preserved IGF‐1 given his hypothyroidism. Cortisol production was not evaluated systematically in our kindred; however, transient neonatal hypocortisolism has also been reported in IGSF1 deficiency.^9^ In this context, it is noteworthy that 3a exhibited neonatal hypoglycaemia; although this is a recognized phenomenon in babies born large for gestational age, we cannot exclude transient cortisol deficiency in the neonatal period although his cortisol production was normal when evaluated in childhood.

Detection of CeCH by neonatal screening requires the inclusion of thyroxine (T4) in the CH screening programme, as in the Netherlands, where an algorithm including TBG enables detection of permanent neonatal central CH.^1^ In keeping with current recommendations, our data support early initiation of levothyroxine replacement in IGSF1 deficiency, as sequelae attributable to hypothyroidism were evident in 70% affected males.^9^ Additionally, associated pubertal delay and GH deficiency may be amenable to treatment following endocrine diagnosis. These observations mandate multigenerational genetic and biochemical ascertainment in all members of IGSF1 deficient kindreds born in countries such as Ireland and the UK, where CH screening programmes fail to detect CeCH neonatally, as well as in adults born prior to the implementation of the screening in countries operating T4‐based methods. This may be a significant undertaking, spanning several healthcare jurisdictions. However, we also highlight the discordance of marked biochemical central hypothyroidism with apparent physiological euthyroidism in some affected individuals, suggesting that formal studies are needed to investigate the benefits of levothyroxine therapy in apparently asymptomatic adults, and to delineate apparent compensatory mechanisms preventing overt tissue hypothyroidism in such individuals.
