# Supplementary material for: A novel IGSF1 mutation in a large Irish kindred highlights the need for familial screening in the IGSF1 deficiency syndrome
Source: Clin Endocrinol (Oxf). 2018 Oct 1;89(6):813–23. doi: 10.1111/cen.13827 (PMC6282842; doi:10.1111/cen.13827)
Supplement: Supplementary file 1 [file CEN-89-813-s001.docx]

**Supplementary Table S1 – Baseline Endocrinology - Hemizygotes**

| Case ID | Age yrs | Prolactin  (NR, mU/L) | IGF1  (NR, nmol/L) | FSH  (Adult  1-10.1U/L) | LH  (Adult  1.5-6.3 IU/L) |
| --- | --- | --- | --- | --- | --- |
| 1a | 75.5 | 124 (90-320) | 25.7 (8.5-30.7) | 11.1 | 3.8 |
| 1b | 73.4 | 271 (45-375) | 19.1 (12.7-29.3) | **24.8** | **9.6** |
| 1c | 69.1 | **27** (45-375) | 18.8 (12.7-29.3) | 9.7 | 3.0 |
| 3h | 13.3 | 115 (45-375) | **15.8** (18.2-69.8) | 2.6 | 0.6 |
| 3i | 12.1 | 101 (45-375) | 16.9 (16.5-65.3) | 3.6 | 0.5 |
| 3a | 7.4 | **<10** (90-320) | 13.3 (7-40) | 1 | <1 |
| 3c | 2.3 | **79** (90-320) | 13.8 (3.6-41.1) | NA | NA |
| 3d | 4.6 | 92 (90-320) | 9.3 (3.6-41.1) | NA | NA |
| 3e | 2.6 | **35** (90-320) | 5.3 (1.7-27.6) | NA | NA |
| 3g | 5 wks | 252 (90-320) | 8.5 (1.7 - 27.6) | 3 | 4 * |

**A**bsolute values for IGF-1, Prolactin, FSH and LH are shown with normal ranges defined in brackets. *3g samples FSH/LH taken at 5 weeks of age in mini-puberty Abnormal values are highlighted in bold.

**Supplementary Table S2 – Baseline Endocrinology - Heterozygotes**

| Case ID | Age (Years) | PRL (100-500U/L) | IGF-1 (nmol/L) |
| --- | --- | --- | --- |
| 1d | 66 | 218 (59-619) | **29.5** (11.8-28.6) |
| 2a | 44.8 | 132 | **32.7** (7.7-28.2) |
| 2b | 43.8 | **31** | NA |
| 2c | 42.6 | 171 | 19.8 (7.7-28) |
| 2d | 39.9 | 382 | 11.1 (8.5-30.7) |
| 2e | 37.3 | **68** | 25.7 (8.5-30.7) |
| 2f | 45.7 | 137 (59-619) | 21.7 (12.6-35.5) |
| 2g | 44.6 | 241 (59-619) | 23.4 (12.6-35.5) |
| 2h | 33.9 | 398 | 26.3 (9.6-31.8) |
| 3b | 6.2 | **45** (90-320) | 20.7(5.1 - 48.2mmol)**^*^** |
| 3f | 1.0 | 199 | 6.5 (2.1-23.1) |

Absolute values for IGF-1, and Prolactin are given, with normal ranges defined in brackets. ^*^ Taken aged 9.1 years Abnormal values are highlighted in bold.

**Supplementary Table S3: TRH Test basal and peak values**

|  | TSH (mU/L) | | FT4 (pmol/L) | | | FT3 (pmol/L) | | | PRL (mU/L) | |
| --- | --- | --- | --- | --- | --- | --- | --- | --- | --- | --- |
| Case ID | Basal | Peak | Basal | Peak | Increment (% Basal) | Basal | Peak | Increment (% Basal) | Basal | Peak |
| 1a | 1.53 | 9.08 | 7.3 | 9.5 | 30.1 | 3.9 | 4.4 | 12.8 | 145 | 767 |
| 3a | 3.94 | 29 | 10.3 | NA | NA | 5.6 | NA | NA | <10 | 46.4 |
| 3c | 2.47 | 23.2 | 9.1 | NA | NA | 5.0 | NA | NA | 145 | 402 |
| 3d | 2.34 | 13.01 | 9.7 | 10.6 | 9.3 | 4.6 | 5.5 | 19.6 | 252 | 1088 |
| 3e | 1.14 | 6.45 | 10.4 | 11.5 | 10.6 | 5.2 | 6.5 | 25.0 | 41 | 214 |
| 3h | 0.36 | 7.17 | 9.2 | 10.3 | 12.0 | 4 | 4.8 | 20.0 | 129 | 944 |
| 3i | 1.42 | 12.9 | 10.2 | 12.4 | 21.6 | 4.3 | 5.7 | 32.6 | 94 | 683 |

Basal reference ranges: TSH (mU/L) 0.3-5.5, FT4 (pmol/L) 10-19.8 1a, 3h, i, 12-22 3a, c, d, e, FT3 (pmol/L) 3.5-6.5 1a, 4.6-7.2 3h, i, 3.1-6.8 3a, c, d, e PRL 90-320 3a, c, d, e, 45-375 1a, 3h, i
